# Supplementary figures and images for: In silico screening of chalcones and flavonoids as potential inhibitors against yellow head virus 3C-like protease
Source: PeerJ. 2023 Apr 24;11:e15086. doi: 10.7717/peerj.15086 (PMC10135407; doi:10.7717/peerj.15086)

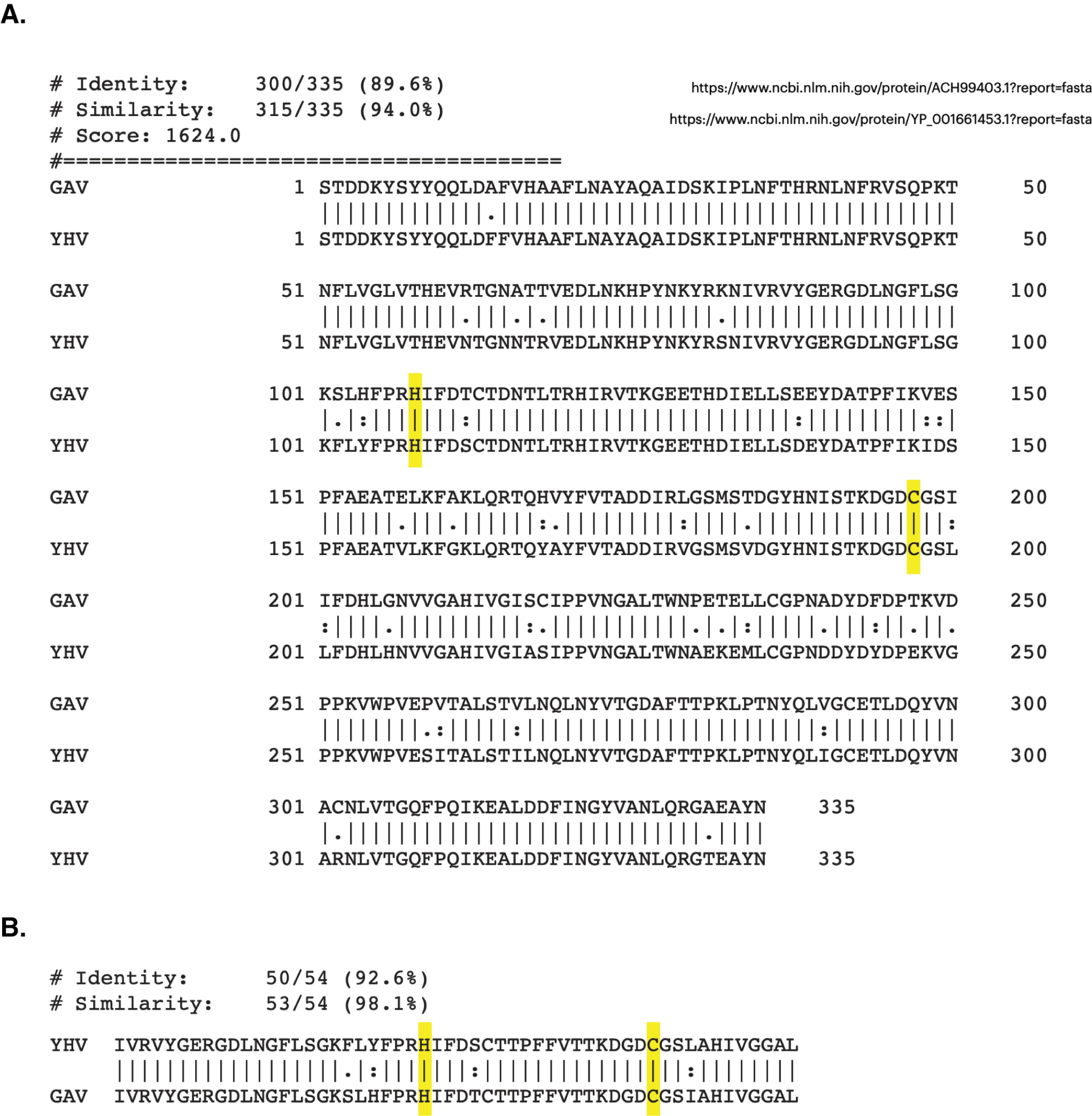

Supplement: Supplemental Information 1 — Quality scores, local distance, and predicted alignment error of YHV (A) and GAV (B) 3CLpro top 5 models created by AlphaFold v.2.0. [file peerj-11-15086-s001.png]

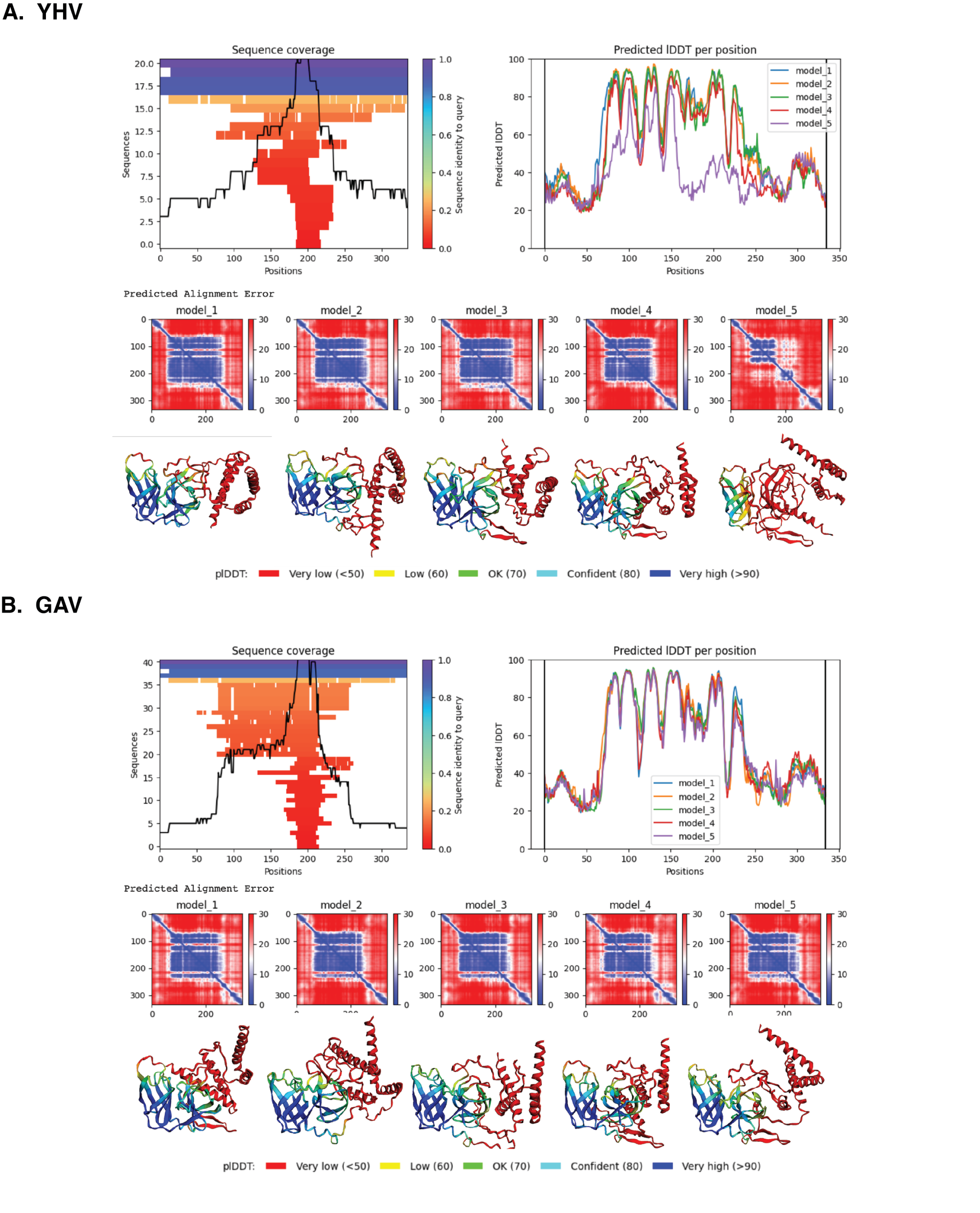

Supplement: Supplemental Information 2 — Amino sequence alignment of YHV and GAV 3CLpro for whole protein (A) and binding pocket (B). [file peerj-11-15086-s002.png]

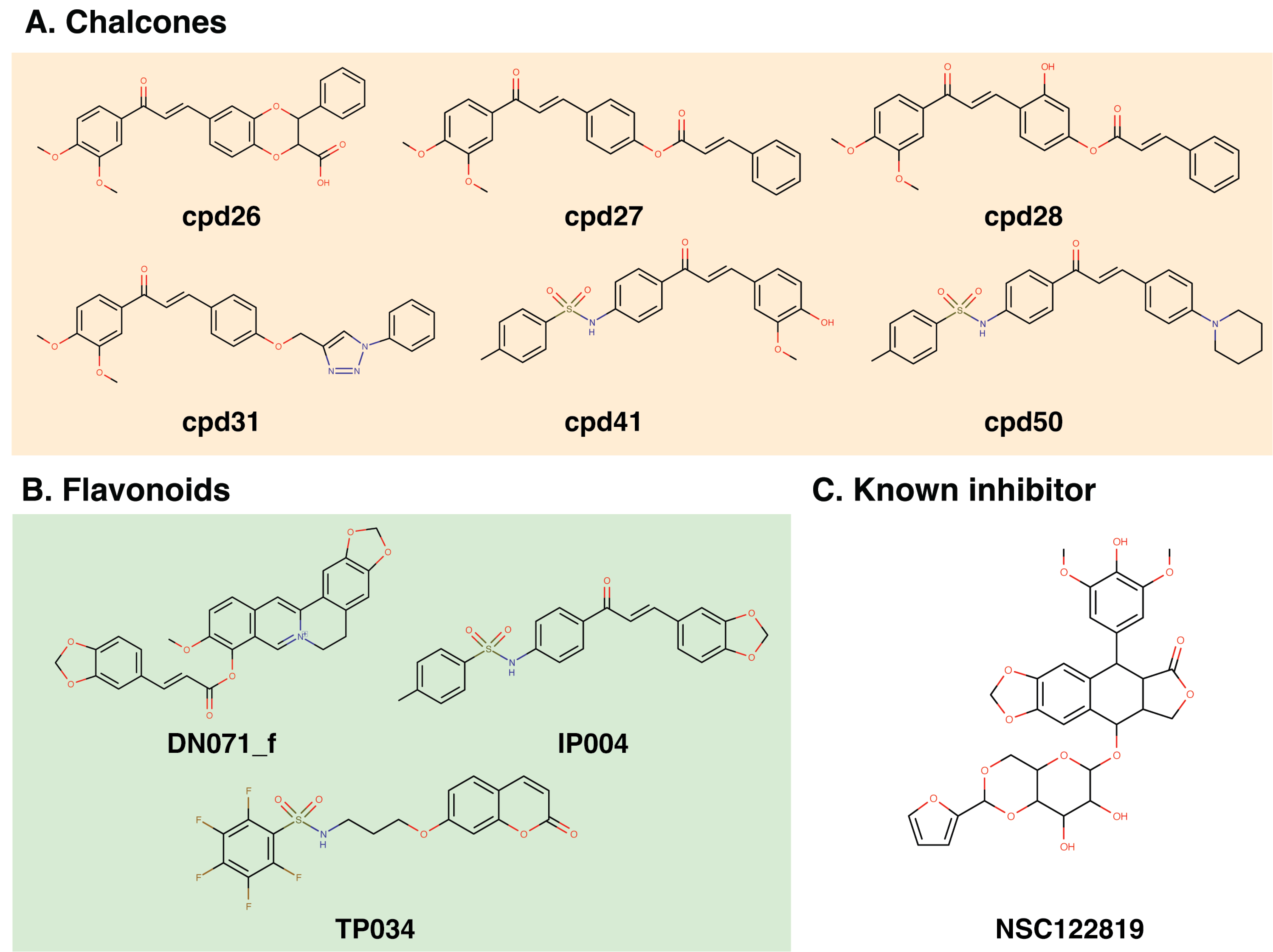

Supplement: Supplemental Information 3 — (A) six chalcones, (B) three flavonoid compounds, and (C) the known YHV inhibitor, namely NSC122819. [file peerj-11-15086-s003.png]

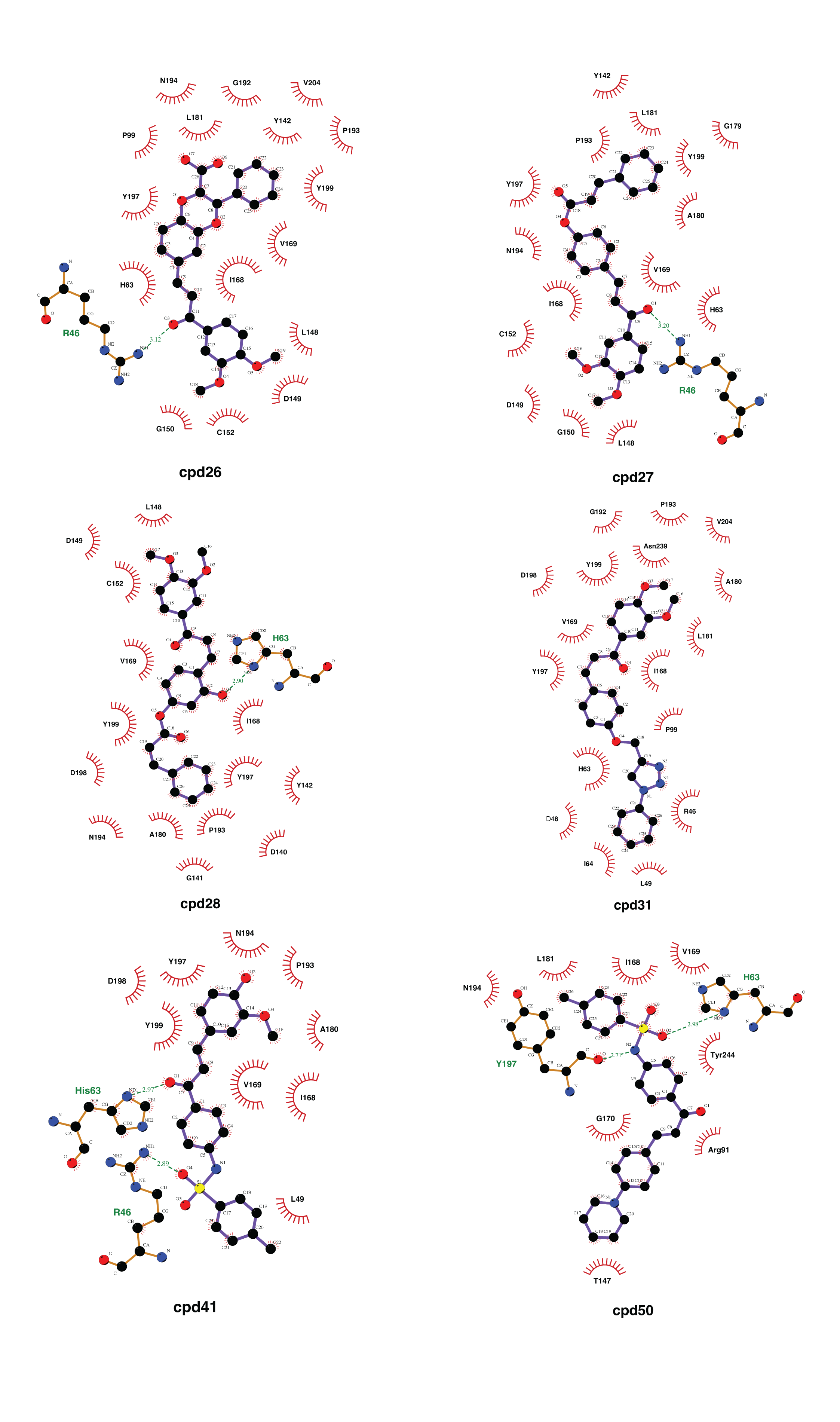

Supplement: Supplemental Information 4 — Two-dimensional analysis of interaction between YHV 3CL pro and 6 chalcone candidates, cpd26, cpd27, cpd28, cpd31, cpd41, and cpd50. Hydrogen bonds are represented by green dash line between atoms involved. Hydrophobic interactions are represented by a red arc with radiating spikes. [file peerj-11-15086-s004.png]

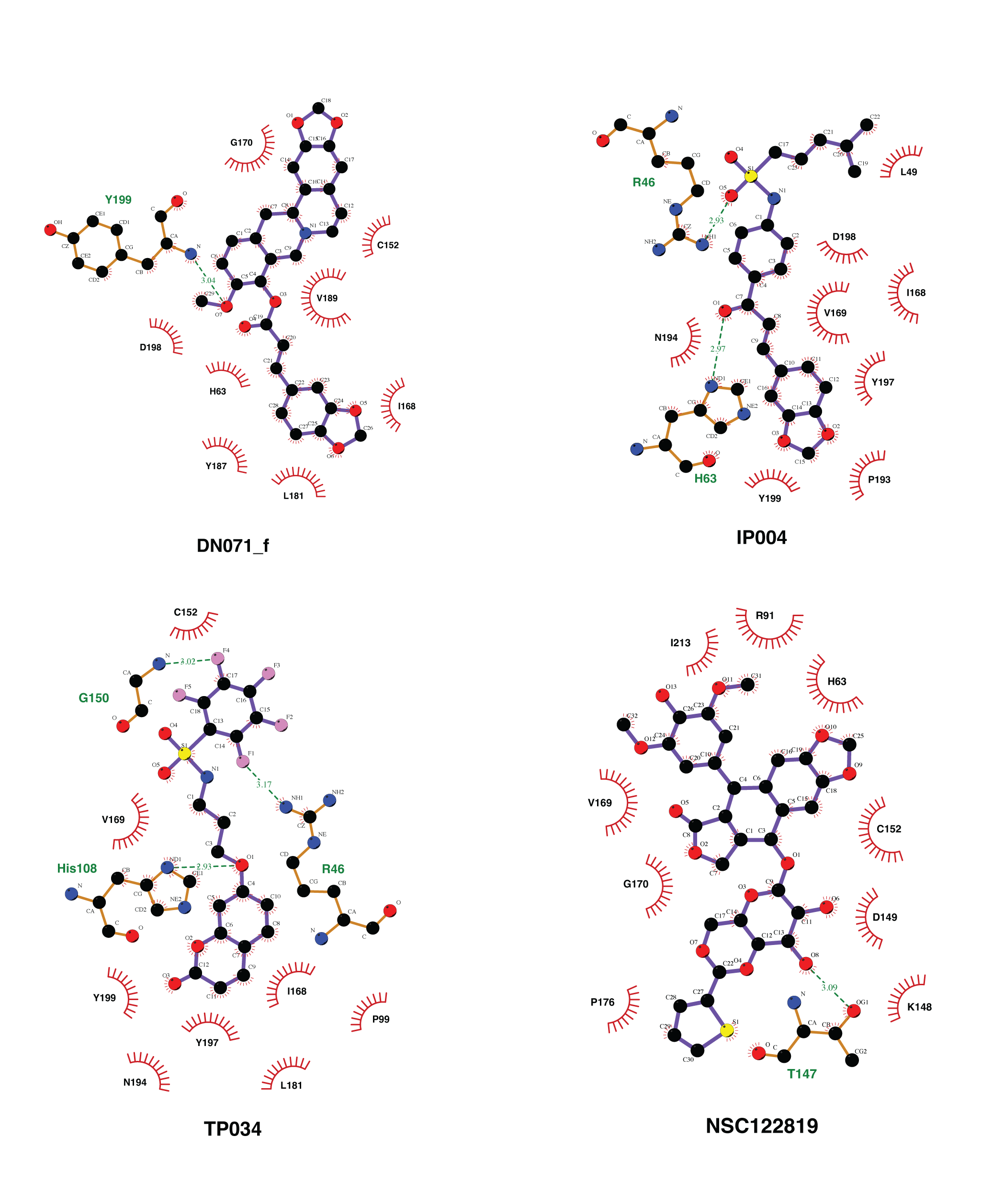

Supplement: Supplemental Information 5 — Two-dimensional analysis of interaction between YHV 3CL pro and 3 flavonoid candidates, DN071_f, IP004, TP034, and the known inhibitor NSC122819. Hydrogen bonds are represented by green dash line between atoms involved. Hydrophobic interactions are represented by a red arc with radiating spikes. [file peerj-11-15086-s005.png]

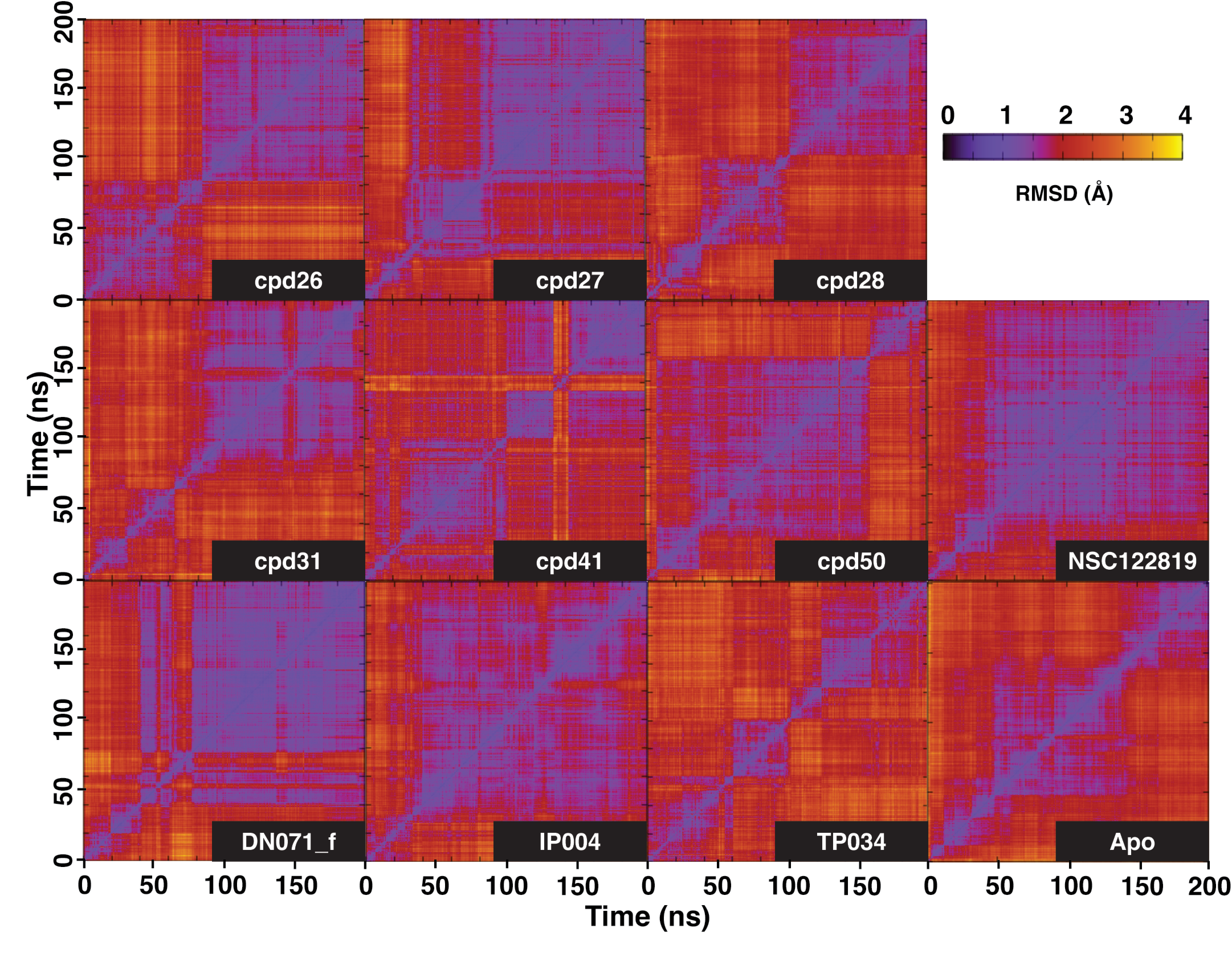

Supplement: Supplemental Information 6 — 2D RMSD plot of 3CLpro-ligand complex and apo-form. The purplish area represented time of the simulation where the system exhibits low RMSD compared to other timepoint, indicating stability of the system. [file peerj-11-15086-s006.png]

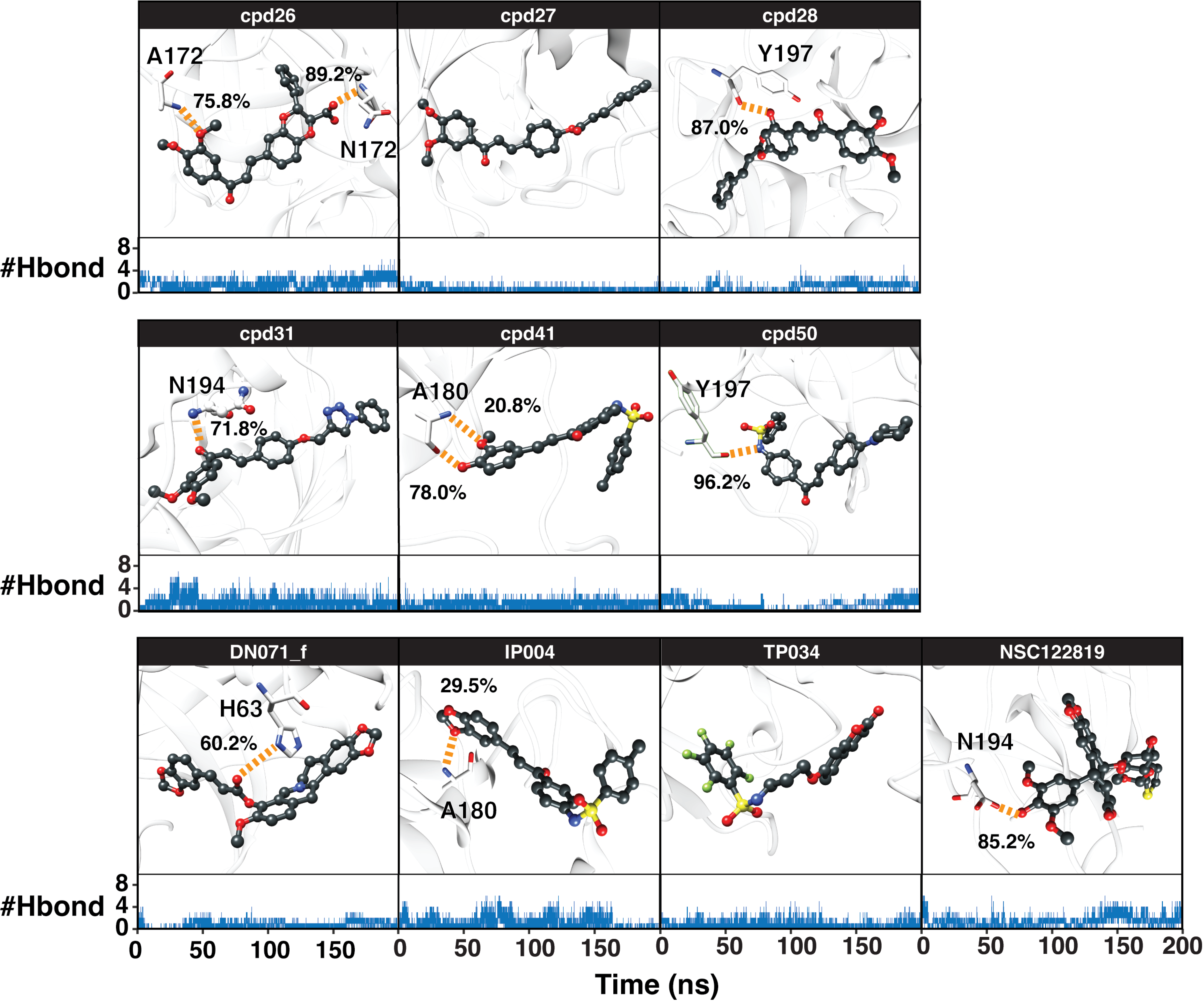

Supplement: Supplemental Information 7 — Percentage of hydrogen bond occupation between the screened compounds and YHV 3CLpro residues (orange dashed line), where the number of hydrogen bonds (#Hbond) upon the simulation time is shown below. [file peerj-11-15086-s007.png]

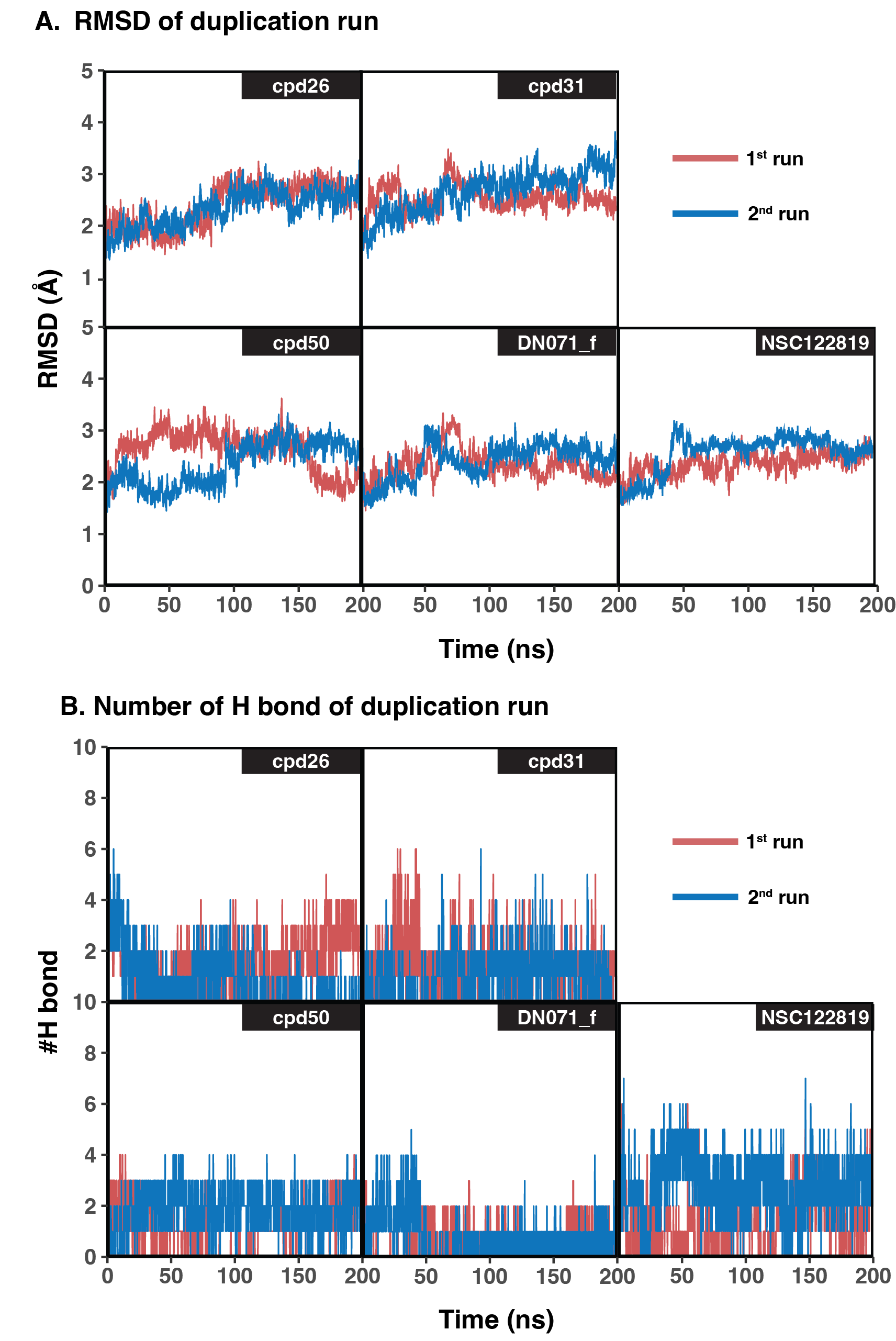

Supplement: Supplemental Information 8 — (A) RMSD result of original (1st run) compared to duplicated simulations (2nd run) of the best four compounds. (B) Number of Hydrogen bond (#H bond) of original and duplicated simulations of the best four compounds. [file peerj-11-15086-s008.png]
